# Spontaneous Rupture of an Infected Pelvic Mesenteric Cyst With Features Suggestive of a Pseudocyst Presenting With Peritonitis

**DOI:** 10.1002/ccr3.73894

**Published:** 2026-11-10

**Authors:** Raya Al Shaaibi, Najah Al Aufi, Ahmed Elsayed, Hossam Elghadban

**Affiliations:** ^1^ Department of General Surgery Ibra Hospital North Al Sharqiyah Sultanate of Oman

**Keywords:** *Escherichia coli*, laparoscopy, mesenteric cyst, peritonitis, rupture

## Abstract

Suspect rupture of a previously documented pelvic mesenteric cyst when acute abdominal pain and new free fluid develop, even if CT no longer clearly shows the cyst. Prompt surgical source control is essential; residual cyst wall warrants clinical and radiological surveillance.

## 1 Introduction

Mesenteric cysts are rare and can develop at any site along the mesentery. The term mesenteric cyst covers several different lesions, including cysts of lymphatic, mesothelial, enteric, and urogenital cysts. The clinical presentation and imaging features often overlap, so the final diagnosis usually depends on the operative findings regarding the origin and histopathology [1, 2, 3, 4, 5, 6].

Most mesenteric cysts are found incidentally or after investigation for vague symptoms like abdominal discomfort, distension, or a palpable mass. Sometimes, they present with acute symptoms such as hemorrhage, infection, torsion, obstruction, or rupture. Rupture with peritonitis is especially uncommon [4, 7]. A pseudocyst is characterized by a fibrous wall without a true epithelial or endothelial lining, and its contents may be serous, hemorrhagic, chylous, or purulent [1, 2].

We reported a large pelvic–mesenteric cyst that had enlarged over two years before rupturing and causing peritonitis. At surgery, the lesion was closely related to the sigmoid mesentery and mesorectum. The case is reported according to the SCARE guidance [8].

## 2 Case History/Examination

A 42‐year‐old woman presented with two days' history of lower abdominal pain, more in the right iliac fossa, and fever with no nausea or vomiting. She had no documented medical comorbidity. Two years earlier, a right pelvic cyst measuring approximately 9 × 9 × 17 cm had been considered ovarian in origin. Further characterization was requested, but she was lost to follow‐up.

She appeared to be in pain. The abdomen was not distended. A reducible paraumbilical hernia with an approximately 3‐cm palpable defect and overlying erythema was present. She had generalized abdominal tenderness with rebound. Her temperature was 38°C; heart rate and blood pressure were normal.

## 3 Differential Diagnosis, Investigations and Treatment

Cystic lymphangioma is an important differential diagnosis. Recent adult reports demonstrate variable symptoms and show that rupture can cause acute peritonitis [4, 6, 9, 10]. In the 2024 report by Kharkhach, the diagnosis depended on dilated lymphatic channels lined by flattened cells and D2‐40 positivity [4]. Those findings were not described in our patient. A peritoneal inclusion cyst is another radiologic differential in reproductive‐age women, in particular after pelvic surgery or inflammation; those lesions usually entrap the ovary within multiloculated fluid and are lined by mesothelium. However, there is no ovarian entrapment and no history of prior pelvic surgery in our patient.

Laboratory testing showed a marked inflammatory response despite a normal total white‐cell count. Selected results are summarized in Table 1.

**TABLE 1:** Laboratory findings at acute presentation, with institutional reference intervals.

Test	Result	Reference interval
White‐cell count	8.51 × 10^3^/μL	2.4–9.5
Neutrophils	7.99 × 10^3^/μL	1.0–4.8
Lymphocytes	0.37 × 10^3^/μL	1.2–3.8
Hemoglobin	11.8 g/dL	11.0–14.5
Platelets	138 × 10^3^/μL	150–450
C‐reactive protein	445.25 mg/L	0–5
Lactate	1.63 mmol/L	0.55–2.2

## 4 Imaging

Two days before the acute presentation, pelvic ultrasonography showed an 18 × 15 × 8 cm right‐sided, thin‐walled, encapsulated fluid collection with low‐level internal echoes and thin septations. No solid component or calcification was seen. Minimal free fluid was present.

On the day of acute presentation, contrast‐enhanced CT showed moderate pelvic free fluid and mildly thickened, enhancing peritoneal reflections, suggesting peritonitis. The appendix measured 9 mm and contained proximal oral contrast without surrounding inflammation. The cyst's origin and rupture were not confidently identified. The previously documented large cyst was no longer clearly visualized as a discrete lesion, consistent with collapse and decompression after rupture.

## 5 Treatment

Peritoneal signs, markedly elevated C‐reactive protein, free pelvic fluid, and CT findings concerning for peritonitis prompted urgent diagnostic laparoscopy. Perioperative antimicrobial treatment comprised piperacillin–tazobactam (Tazocin) for a total of five days.

Laparoscopy revealed turbid blood‐stained fluid in the pelvis, perihepatic and perisplenic spaces, and between bowel loops (Figure 1). A ruptured cyst had remnants attached to the suprapubic region (Figures 2, 3, 4). The lesion bordered the right sigmoid mesentery and extended toward the mesorectum. Accessible wall was dissected lateral to the bowel using an advanced energy device. The cyst wall was adherent to the sigmoid colon and rectum. Complete excision was considered unsafe because dissection of the adherent wall risked bowel injury. A minimal amount of wall attached to the colon was therefore left in situ. Rectal insufflation after intraluminal methylene blue showed no air or dye leak. Tissue and fluid were sent for histopathology, cytology, and microbiology. Drains were placed in the pelvis, right subhepatic space, and left subphrenic space. The three drains were intended to drain the contaminated pelvic and bilateral upper abdominal compartments and reduce the risk of postoperative fluid collections. The paraumbilical defect and left lumbar port fascia were closed primarily without mesh because of contamination. The paraumbilical hernia was an incidental finding and was not considered the source of peritonitis.

**FIGURE 1:**
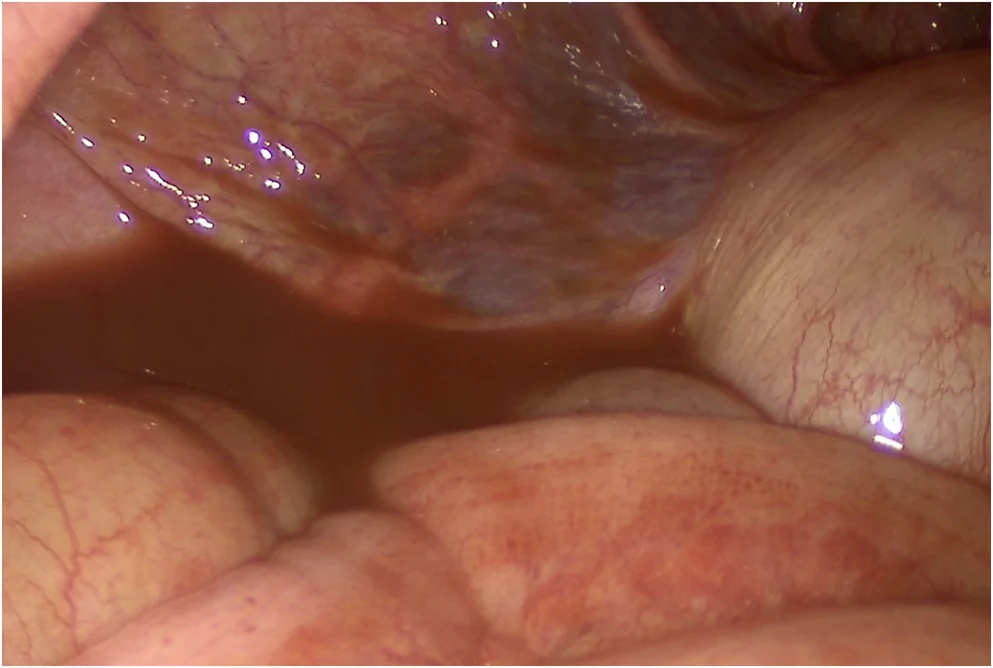
Operative view showing turbid blood‐stained pelvic fluid.

**FIGURE 2:**
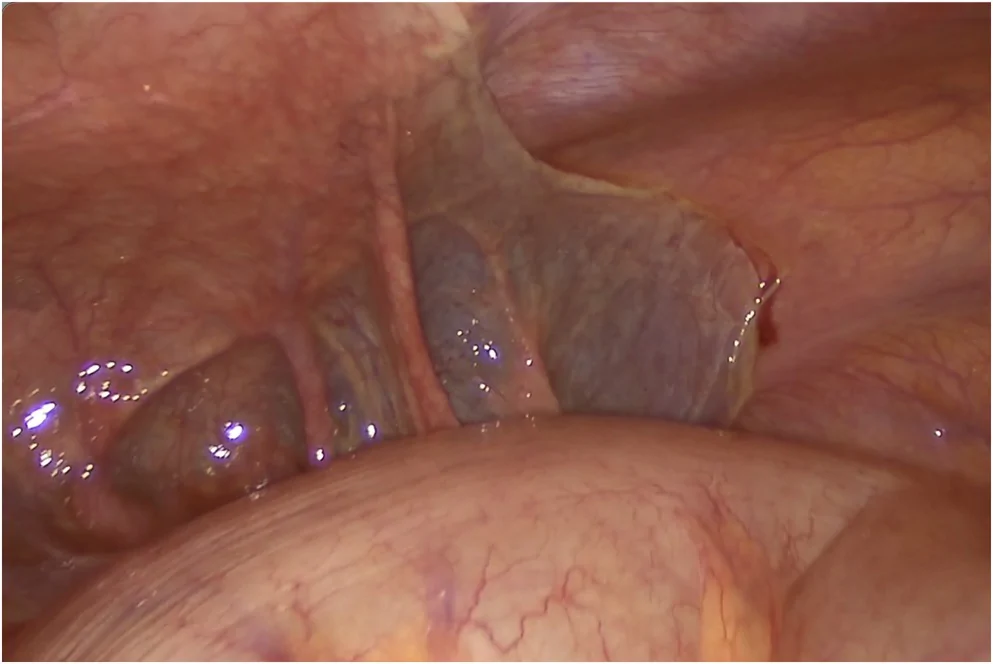
Operative views of the ruptured cyst and intraperitoneal fluid. This figure shows wall attached to the anterior abdominal wall.

**FIGURE 3:**
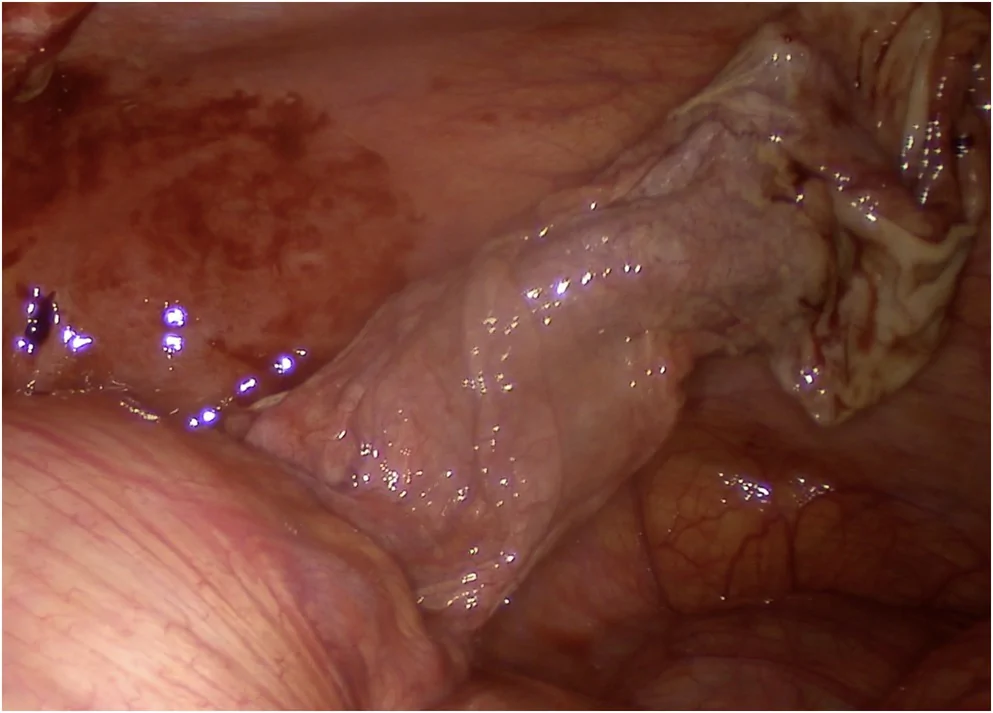
Operative view showing collapsed mesenteric cyst remnants.

**FIGURE 4:**
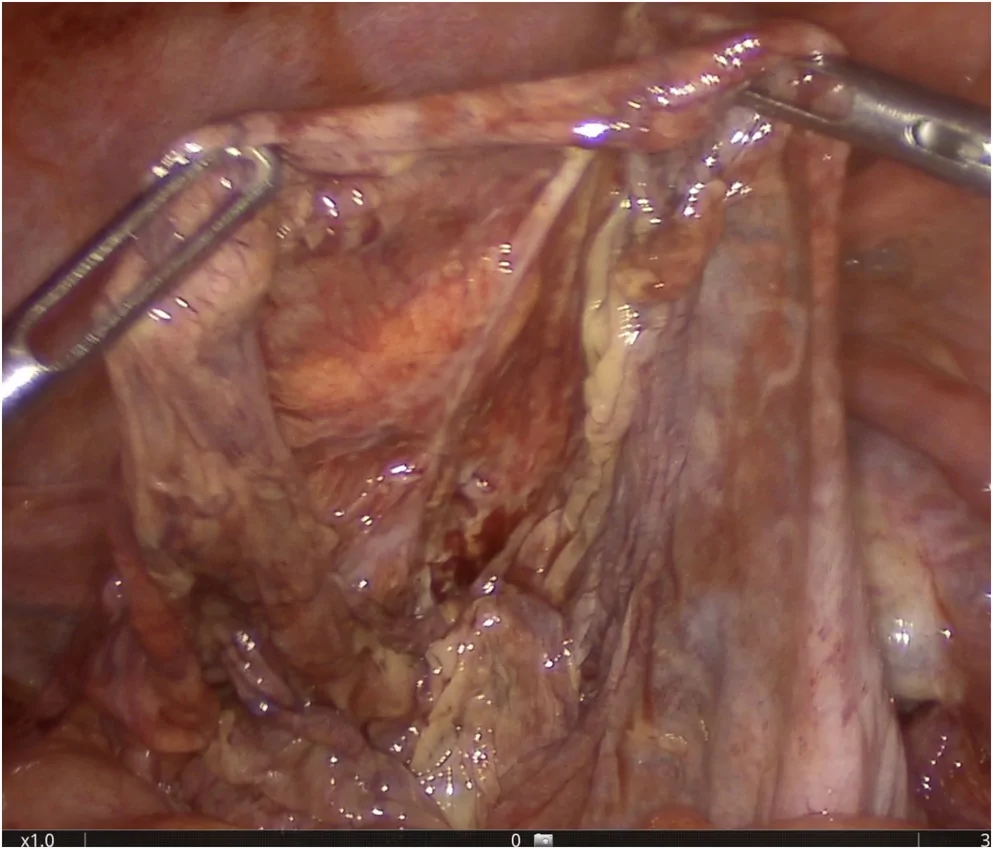
Operative view showing the ruptured mesenteric cyst wall.

## 6 Pathology, Cytology, and Microbiology

Gross examination showed multiple tan soft‐tissue fragments measuring 9 × 1.5 × 1 cm in aggregate; no intact cyst, solid area, or mass was identified. Microscopically, the fragments consisted of congested fibrocollagenous tissue, mainly surfaced by fibrinous exudate and focally by reactive mesothelial cells. The stroma contained a marked mixed inflammatory infiltrate of lymphoplasmacytic cells and neutrophils, with focal microabscess formation. No epithelial lining, granuloma, or atypical cell population was identified. Periodic acid–Schiff and Grocott methenamine silver stains were negative for fungal organisms.

Fluid cytology showed numerous inflammatory cells, predominantly neutrophils, with reactive mesothelial cells that were calretinin positive and Ber‐EP4 negative. No malignant cells were identified. Culture grew *E. coli* resistant to amoxicillin but susceptible to amoxicillin–clavulanate, ceftazidime, ceftriaxone, ciprofloxacin, gentamicin, and trimethoprim–sulfamethoxazole.

## 7 Conclusion and Results (Outcome and Follow‐Up)

Postoperatively, pain and fever resolved. The drains were removed, and the patient was discharged in good condition. At one‐month follow‐up, abdominal ultrasonography showed minimal pelvic free fluid and no visible cyst. No longer‐term follow‐up is reported.

An infected pelvic mesenteric cyst may rupture and cause bacterial peritonitis. Acute abdominal pain and new free fluid in a patient with a previously documented cyst should prompt consideration of rupture, even when CT does not confidently establish its source. Surgery provides source control and tissue for examination. In this case, histology suggested a pseudocyst, but fragmented, markedly inflamed tissue prevented definitive classification. Because residual wall remained, continued clinical and radiological surveillance is advisable.

## 8 Discussion

Mesenteric cyst is an anatomical term rather than a specific diagnosis. These lesions may be lymphatic, mesothelial, enteric, urogenital, or germ‐cell in origin; some are non‐pancreatic pseudocysts. Histology is therefore important for identification. Lymphatic lesions usually contain endothelial‐lined spaces and may stain with D2‐40, while mesothelial cysts have a mesothelial lining. Pseudocysts, in contrast, have a fibrous wall without a true lining and may present with inflammation, hemorrhage, or necrosis [1, 2, 3, 5].

The operative findings supported a pelvic mesenteric or intraperitoneal cyst. The inflamed fibrocollagenous wall without a demonstrable epithelial lining suggested a non‐pancreatic pseudocyst, but this subtype could not be established definitively. Examination was limited to fragmented, markedly inflamed tissue. Reactive mesothelial cells may have represented the adjacent peritoneal surface, and severe inflammation may have destroyed a pre‐existing epithelial or endothelial lining. We therefore describe the lesion as an infected ruptured pelvic mesenteric cyst with features suggestive of a pseudocyst.

Infection and rupture are unusual complications of mesenteric cysts and may cause peritonitis and sepsis. Recent reports describe free intraperitoneal fluid and inflammatory changes with chylous chemical peritonitis or frank infection [4, 7]. Although the total white‐cell count was normal, CRP exceeded 445 mg/L, with neutrophilia and marked lymphopenia. Turbid fluid, mural microabscesses, and *E. coli* growth confirmed infection.

The imaging sequence illustrates the diagnostic challenge. Ultrasonography two days before acute presentation showed a large encapsulated pelvic cyst with minimal free fluid. CT at acute presentation showed free fluid and peritoneal inflammation, while the previously documented large cyst was no longer clearly visualized as a discrete lesion. This change was consistent with collapse and decompression after rupture, subsequently supported by operative identification of a ruptured cyst.

Complete excision is preferred for a symptomatic mesenteric cyst provided it can be performed safely, together with drainage of infected fluid. This provides the best diagnostic specimen and reduces recurrence [2, 3, 5, 6]. The laparoscopic or open approach depends on cyst size and location, patient condition, contamination, involvement of bowel or major vessels, and expertise. Bowel resection may be necessary. When complete excision is unsafe, partial excision or marsupialization may be considered, but residual wall requires surveillance because recurrence is possible [7].

Strengths include serial imaging, operative documentation, microbiology, cytology with immunophenotyping, and histology with fungal stains. The principal limitation is incomplete etiologic classification because rupture left only a fragmented wall for examination. Histopathology microscopy images could not be obtained because the specimens were processed at another hospital. Follow‐up was limited to one month, which does not exclude later recurrence.

## Author Contributions

**Raya Al Shaaibi:** conceptualization, writing – original draft, methodology, resources. **Najah Al Aufi:** investigation, validation. **Ahmed Elsayed:** validation, investigation. **Hossam Elghadban:** investigation, validation, supervision.

## Funding

The authors have nothing to report.

## Ethics Statement

The authors have nothing to report.

## Consent

Written informed consent from the patient was obtained according to journal guidelines.

## Conflicts of Interest

The authors declare no conflicts of interest.

## Data Availability

All data supporting this case report are included in the article. Additional de‐identified information may be available from the corresponding author on reasonable request, subject to institutional and ethical requirements.
